# Door to Door Survey and Community Participation to Implement a New County Mosquito Control Program in Wayne County, North Carolina, USA

**DOI:** 10.3390/ijerph6082150

**Published:** 2009-07-31

**Authors:** Amanda Grantham, Alice L. Anderson, Timothy Kelley

**Affiliations:** 1 Wayne County Health Department, Goldsboro, NC 27534, USA; E-Mail: Amanda.Grantham@mail.wayne.nc; 2 East Carolina University, 3403 Belk, Greenville, NC 27858, USA; 3 East Carolina University, 3405 Belk, Greenville, NC 27858, USA; E-Mail: kelleyt@ecu.edu

**Keywords:** community health, mosquito control, community survey

## Abstract

Community involvement in mosquito management programs provides more sustainable and effective organization and service. A door to door survey in Wayne County, NC carried out by student volunteers, resulted in 60 household responses. Residents had not previously experienced outreach from the county (88%), and 95% of them thought the student door to door survey was an effective form of outreach. One third of the residents thought mosquitoes were severe where they lived, but only 9% thought they had any containers in their yard that might breed mosquitoes. Only 15% of the residents were concerned about mosquito borne diseases. These responses provide evidence that outreach and education on mosquito control and diseases were necessary steps for future mosquito control community planning.

## 1. Introduction

**Figure 1.**
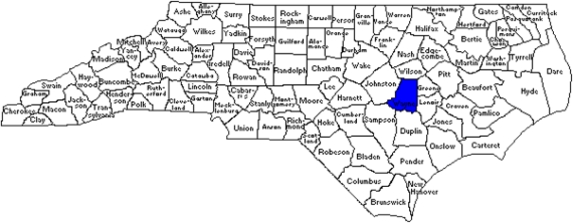
Location of Wayne County in North Carolina, USA.

Mosquitoes in the US are economic pests and can potentially spread harmful diseases, such as West Nile meningoencephalitis, Eastern Equine encephalitis and St. Louis encephalitis. The management of these disease vectors must include control of mosquito populations in and around the individual homes in a community in order to reduce human exposure to disease. This study took place in Wayne County, North Carolina, which is home to about 115,000 residents, included in seven municipalities. There is an average of 86 homes per square mile, in a county 120–145 feet above sea level. Figure 1 shows a map with the location of Wayne County in North Carolina, USA, located in the Neuse River watershed.

Until recent years Wayne County’s mosquito control program had consisted only of complaint forms available online. Because of an increased number of citizen complaints, the county proposed to plan and implement a more comprehensive program in the 2007–2008 fiscal year. In this new effort, the county planned to educate the public on how to reduce mosquito populations around their homes, and to implement other mosquito prevention measures, including a mosquito surveillance program, and integrated efforts between the county and its seven municipalities.

Research suggests that vector control programs with community participation have significant and lasting impacts on vector density, and are more cost-effective than vertically structured programs. In addition, these types of programs have been reported to readily integrate with other health or development programs, promote an enduring sense of pride in home and community, and make use of politically viable vector control strategies [1,2].

Traditional vector control programs, in which governments traditionally accepted the responsibility for mosquito control and communities simply depended on those services have tended to lack sustainability. When the government was no longer able to support vector control services, the communities were back where they started, having made no progress in managing vector populations [3]. Bryan *et al*. [1] and Wilson *et al*. [4] both concluded that a program including community participation has proven to be the most sustainable method of vector control. Community participation was shown to have inherent value because of its positive effect on social relationships and community solidarity. It was seen to be a dynamic process that resulted in accrued benefits for public health that exceed most vector control program goals and persisted well beyond program termination [1].

A vital part of community-based vector control programs is an investigation of local perceptions of disease and health, disease transmission, the control and prevention of disease, and behavior related to the use of health services. Community perceptions obtained from survey information can then be incorporated into a control project that is specific for community needs [3,5].

Some examples of new programs with community involvement in developing countries are appropriate to compare to minimal mosquito control counties in the US, especially now that budgets are being drastically cut back in many states. A mosquito control program in Merida, Yucatan, Mexico, for example, implemented a “bottom up” (or grassroots) type of community-based *Aedes aegypti* control program, beginning in 1989 [3]. After much research and development, the project “demonstrated that a community-based communication program aimed at larval production site elimination or control could be effective in changing behavior and reducing larval production sites” [3]. In Tanzania, use of larvicides in a large-scale community-based program reduced malaria infection. Reducing mosquito populations throughout the season was the key [6].

“Top down” methods by contrast, can be very expensive and have no long-term effect. In North Carolina, though mosquito control programs have been present for at least 35 years, many communities continue to be unprepared for heavy mosquito seasons, particularly following storms. Many communities must then depend on the state to provide emergency mosquito control and have less input into the process, as occurred after Hurricane Floyd [7].

Mosquito control in the United States in general has evolved from a reliance on insecticide application for control of adult mosquitoes to an integrated pest management programs that include surveillance, larvacide, source reduction and biological control in addition to public relations and education [8]; however, in many vulnerable communities there are still no programs or very basic “spray on demand” top down programs.

The planned mosquito control program at the Wayne County Health Department in this research project concentrated on the “bottom up” sustainable approach to create a program, and had three main goals. The first was to create an efficient method for control of mosquito populations in Wayne County to reduce the likelihood of the spread of disease and the annoyance of mosquito bites. The second was to plan for a better integration of mosquito control across municipalities, Seymour Johnson Air Force Base, and the Wayne County Government. The third was to educate and include the citizens through outreach and technology. This community participation included individual compliance with recommended behavioral changes [1], such as eliminating container breeding habitats around the home.

Public education is also an important component of homeowner backyard source reduction [8]. Many counties, including Wayne County, NC and state mosquito control agencies, have public school education programs that teach children what they and their families can do to prevent mosquito breeding around their homes.

In order to involve individual residents and communities in vector control, more information is usually needed about the public’s knowledge, attitudes, and behavior regarding mosquitoes and the diseases they transmit [9,10]. Students in many community schools in North Carolina are now often required to engage in community service projects during their senior year. This project therefore put these two elements together in a community involvement health education project concerning a new community involved mosquito and vector control program.

### Wayne County Plan

The mosquito control program implementation in Wayne County initially had seven different components, the first of which was to the train the staff of the Environmental Health program located in the health department. Two Environmental Health Specialists (EHS) were trained to identify mosquitoes to genus and species. They were also trained to understand the biology and life cycles of different species in terms of their threat to the public. One EHS acquired a public health pesticide applicator’s license so that larvacide could be used. Finally, staff were also trained and educated to act as mosquito control Public Information Officers for the County.

A second technical component of the plan, which is not a part of this report, was the use of GPS mapping. Third, training and mobilization of the community was initiated. The other components of the seven were sub parts of this training and mobilization. The county arranged education programs for Homeowners Associations in Wayne County. The message was that citizens could be responsible for insect control using environmentally sound practices on their own property. Communication with the public was an important part of the program implementation. Frequent communication with the public via the county’s website, the county’s local television station, and newspaper were planned to remind the public of the part they could play in reducing mosquito populations. The county wanted to establish an integration effort between the municipalities in Wayne County, as well as with Seymour Johnson Air Force Base, so the county planned to establish a list serve so that each department could communicate with the others regarding spraying areas and schedules, findings, and future efforts. To aid in establishing sustainability of the program, the county created a website that contained mosquito information and was easily available to the public. With this mosquito control program, a system was implemented to ensure that the website was frequently updated and offered important information to the public. When the student intern positions were created, students also volunteered to maintain a personal webpage for their school.

## 2. Survey Methods

A major part of the educational component of this plan was to give high school student interns an opportunity to do surveillance, larvacide application, and public education. The county and a local high school forged an agreement enabling the students to intern with EHS staff for one afternoon a week for a month. The student interns were high school students from a local science and technology school, the Wayne County School of Science and Engineering.

To orient the students, several presentations were given at the school concerning the biology and life cycle of mosquitoes, as well as explanations of the plans for assisting Wayne County with their efforts to implement a mosquito control program.

The main responsibility for the students was to distribute a mosquito knowledge and attitude questionnaire throughout most of the subdivisions in Wayne County, supervised by an Environmental Health Specialist. The students surveyed an area less than two square miles with over 500 homes (well above the 86 homes per square mile county average). Each group of 10–12 students spent one afternoon interning with the Environmental Health Department in April and May of 2008. The results of this survey are the main subject of this report.

The survey was comprised of nine short questions pertaining to the prevention measures that residents were currently taking against mosquito exposure, their current knowledge of mosquitoes and disease transmission, and what county residents would like to see in a mosquito control program. Ethics approval for the survey was obtained from county officials.

High school student interns in this community involvement and mobilization project administered a door-to-door survey in four connecting subdivisions in northern Wayne County. The subdivisions were Lane Tree, North Creek, Chancery and North Pointe. The subdivisions were chosen for this survey by the county administrators because they had a history of mosquito complaints and because they were diverse subdivisions. The tax values of these homes ranged from $100,000 to over $400,000. There was a wide range of socioeconomic level in a relatively small area, which allowed for the reduction of economic status bias in this study.

A packet of education materials was provided to every home that was surveyed. If homeowners were at home during the sampling time of 1:00 PM to 4:00 PM, students asked the homeowner to participate in a short questionnaire to help the county in their efforts for mosquito control. The educational materials were left at the door for homeowners not present. The educational packet consisted of a questionnaire, mosquito information brochure, container breeding habitat survey, and an informational homeowner letter.

The mosquito information brochure was a publication of the North Carolina Department of Environment and Natural Resources (NCDENR) that outlined the life cycle of mosquitoes, included disease information as well as a few prevention tips. A container breeding habitat survey was included for the homeowners to use in evaluating their own property. Lastly, the homeowner informational letter was a letter written by the Environmental Health Director of Wayne County, providing guidelines for personal protection against mosquitoes, guidelines for reducing mosquito breeding areas around the home, and all of the contact information for the Wayne County Environmental Health Department.

Over 300 of the 500 homes in the subdivisions were visited, with the interns covering over 60% of the area in the four days allotted. Sixty questionnaires were completed by homeowners for a 20% response rate. This was well above the 10% response rate the county wished to achieve.

## 3. Results and Discussion

**Figure 2.**
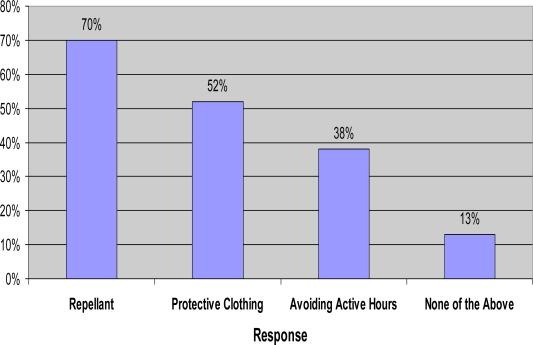
Percent of residents using prevention measures against mosquitoes.

All of the survey answers and response percentages are displayed in Table 1. The first question on the survey stated, “Is this the first outreach on mosquito control you have had (Includes previous newspaper articles and radio information)?” Eighty-eight percent of the residents surveyed answered “yes”, while twelve percent of the residents had previously had an outreach initiative. The second question, “Are you aware of the mosquito control services in Wayne County?” received a 33% response for “yes”, and a 67% response for “no”. Third, “Have you visited the Wayne County Environmental Health mosquito webpage and blog?” had 92% of those surveyed responding “no”, while only 8% responded “yes”. The fourth question inquired “Do you use any of the following mosquito prevention measures- repellant, long sleeves and pants, avoid being outside during dusk or dawn, or none of the above?” Twenty-two percent of those surveyed answered that they just use repellant as a form of protection against mosquito exposure, while seventy percent claim to use repellant overall. Seven percent said that they only wore long sleeves and pants while outside, while fifty-two percent wore protective clothing alone or with another form of prevention. Five percent used the method of avoiding times of highest mosquito activity alone, while thirty-eight percent of people avoided being outside at dusk and dawn overall. Eighteen percent of those surveyed claimed to use all three types of personal protection (Figure 2) against mosquito exposure. Eighteen percent answered that they used “none of the above” measures to avoid mosquito exposure.

**Figure 3.**
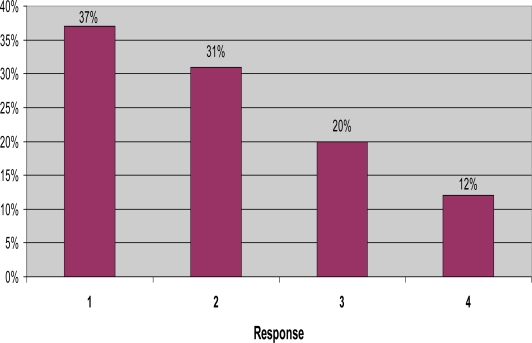
Severity of mosquitoes around the home, on a scale of 1 to 4.

The fifth question on the survey asked “On a scale of 1 (least severe) to 4 (most severe), how severe are the mosquitoes around your home?” Thirty-seven percent of the residents answered “1”, and 31% answered “2”. Twenty percent of those surveyed responded “3”, and only 12% answered “4” (Figure 3). Homeowners can vary on their opinion of what “severe” mosquitoes means, so the results may be somewhat ambiguous.

**Figure 4.**
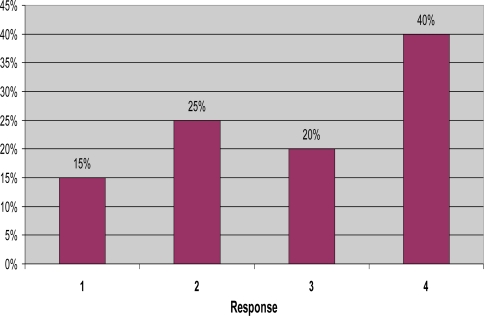
Concern with potential disease, on a scale of 1 to 4.

The sixth question was, “On a scale of 1 (least concerned) to 4 (most concerned), how concerned are you with diseases that mosquitoes may potentially carry?” Only 15% of residents claimed to be concerned only on a level of “1”. A quarter of the population stated “2”, twenty percent “3”, and an overwhelming forty percent answered “4” (Figure 4). Many residents were concerned about potential diseases that are associated with mosquitoes, and are hopefully being stirred to action and utilizing prevention measures.

The seventh question asked “Do you have any containers holding water, or low areas with standing water around your home?” This question was divided closely, with 47% answering “yes”, and 53% responding “no”. The eighth question was, “Did you know that there is a complaint form available online that you can submit to request mosquito management services?” Only 9% of those surveyed answered “yes”, while an overwhelming 91% answered “no”.

The last question of the survey was potentially the most important. It asked, “Do you feel that this type of outreach can be effective?” The 95% “yes” response rate indicated that the county’s efforts were being set in the right direction. Only 5% of the population did *not* feel this type of outreach best suited the general population.

## 4. Discussion

“To be successful, community-based strategies must be flexible and adapted to the local setting because of ecologic, cultural, and social differences between localities” [3].

Overall, the use of interns was very successful. Observational reports from the health department employees indicated that the students were professional in their behavior, and completed the surveying effectively. However, increased pre-survey training is planned for the future to give the students more practice with interviewing techniques. This process could be evaluated for future years. Some of the questions may be slightly reworded in future efforts to be more specific in nature.

After analysis of the survey process, a few county procedural changes were made for future surveys. Most importantly, the county will increase media coverage of the surveying. Many homeowners thought the students were selling or marketing, and tended to avoid them. It is hoped that increased awareness of the students and their efforts to help the county Health Department will in turn increase the number of responses to the surveys. More time was needed for the surveying process than was planned, so students did not have enough time to survey all of the locations. The county will plan for a longer surveying period in the future.

## 5. Conclusions

Assessment of Wayne Co., NC, USA residents in a door to door survey about mosquitoes illustrated that residents were willing to learn about mosquitoes, and that personal outreach efforts by students was viewed very positively. Only thirty-two percent of the community surveyed thought that mosquitoes were severe to moderately severe, but the survey question was somewhat ambiguous. Many more people were concerned about mosquito borne diseases (60%). Of those people, however, only a few knew of the online complaint forms that could be used to obtain county service. These disparities will be addressed in the community involvement surveys in the future, and in the improvement in the level of communication venues, with web pages, TV announcements, radio, and print media. A disappointingly large number of residents reported having containers holding water in their yards, so a continuous effort at reminding people about the mosquitoes they produce was warranted.

The County created a website (http://www.waynegov.com/16581031616394430/blank/browse.asp?a=383&BMDRN=2000&BCOB=0&c=54580&16581031616394430Nav=|231|&NodeID=231) that contained mosquito information and was easily available to the public.

The local EHS are continuing to collect GPS data points for complaints that come in, as well as recording any areas where larvacides were applied, or where a trap was placed. The county is also continuing to increase communication between municipalities in their efforts to jointly control mosquitoes as a county. All of these continuing efforts are expected to have an impact on the mosquito population their annoyance, and their disease vectoring potential.

## Figures and Tables

**Table 1.** Sixty household door to door survey percent results.

Questions regarding mosquitoes and mosquito control				
1	Yes	No		
First outreach	88%	12%		
2	Yes	No		
Aware of services	33%	67%		
3	Yes	No		
Visit webpage	8%	92%		
4	Repellant	Protective clothing	Avoid active hours	None of the above
Preventive actions	70%	52%	38%	13%
5	1	2	3	4
Severity of mosq	37%	31%	20%	12%
(4 worst)				
6	1	2	3	4
How concerned	15%	25%	20%	40%
7	Yes	No		
How containers	47%	52%		
8	Yes	No		
Complaint form found	9%	91%		
9	Yes	No		
Outreach good	95%	5%		

## References

[1] Bryan R Balderrama F Tonn R Dias J Community participation in vector control lessons from chaga's disease Amer. Soc. Trop. Med. Hyg 1994 50 61 71 10.4269/ajtmh.1994.50.61 8024086

[2] Hahn T Hill P Kay B Quy T Development of a framework for evaluating the sustainability of community-based dengue control projects Am. J. Trop. Med. Hyg 2009 80 312 318 19190231

[3] Lloyd L Winch P Ortega-Canto J Kendall C Results of a community based Aedes Aegypti control program in Merida, Yucatan, Mexico Am. J. Trop. Med. Hyg 1992 46 635 642 1621887 10.4269/ajtmh.1992.46.635

[4] Wilson SD Varia M Lior LY West Nile Virus: the buzz on Ottawa residents awareness, attitudes and practices Can. J. Public Health 2005 96 109 13 15850029 10.1007/BF03403672 PMC6975773

[5] Gubler D Aedes aegypti and Aedes aegypti-borne disease control in the 1990’s: top down or bottom up Am. J. Trop. Med. Hyg 1989 40 571 578 2472746 10.4269/ajtmh.1989.40.571

[6] Geissbuhler Y Kannady K Chaki PP Emidi B Govella NJ Mayaguya V Kiama M Mtagiwa D Mshinda H Lindsay SW Tanner M Fillinger U deCastro MC Killeen GF Microbial larvicide application by a large-scale, community-based program reduces malaria infection prevalence in urban Dar es Salaam, Tanzania PLoS ONE 2009 4 e5107 19333402 10.1371/journal.pone.0005107 PMC2661378

[7] Anderson AL Engber B Harrison B Whitt P Newton N Emergency aerial spraying in North Carolina after hurricane Floyd Wingbeats 2000 11 4 5

[8] Rose R Pesticides and public health: integrated methods of mosquito management Emerg. Infect. Dis 2001 7 17 23 11266290 10.3201/eid0701.010103 PMC2631680

[9] Klein R Weller S Zeissig R Richards F Ruebush T Knowledge, beliefs and practices in relation to Malaria Transmission and Vector Control in Guatemala Amer. Soc. Trop. Med. Hyg 1995 52 383 388 10.4269/ajtmh.1995.52.383 7771601

[10] Yasuoka J Magnione TW Speilman A Levins R Impact of education on knowledge, agricultural practices, and community actions for mosquito control and mosquito borne disease prevention in Sri Lanka Am. J. Trop. Med. Hyg 2006 74 1034 1042 16760516

